# Characterization of Enlarged Kidneys and Their Potential for Inducing Diabetes in DEK Rats

**DOI:** 10.3390/biology10070633

**Published:** 2021-07-08

**Authors:** Ayaka Domon, Kentaro Katayama, Takashi Yamada, Yuki Tochigi, Hiroetsu Suzuki

**Affiliations:** Laboratory of Veterinary Physiology, Faculty of Veterinary Science, School of Veterinary Medicine, Nippon Veterinary and Life Science University, 1-7-1 Kyonancho, Musashino-shi, Tokyo 180-8602, Japan; v.o.dl.pa.up.bws@gmail.com (A.D.); katayama@nvlu.ac.jp (K.K.); takashi.y.1118@outlook.jp (T.Y.); ytochigi@nvlu.ac.jp (Y.T.)

**Keywords:** diabetes, animal model, kidney, nephron number

## Abstract

**Simple Summary:**

The worldwide prevalence of diabetes mellitus (DM) in 2020 has been estimated at 463 million patients. About 90% of patients with diabetes have type 2 diabetes mellitus (T2D), caused primarily by insulin resistance and insufficiency. However, the specific etiology of T2D remains unknown. Sodium glucose cotransporter 2 (SGLT2) inhibitors are a novel class of anti-diabetic drugs that act independently of insulin and reduce blood glucose concentrations by inhibiting the reabsorption of glucose at renal proximal tubules. SGLT2 inhibitors have highlighted the role of the kidneys in glycemic control in diabetes. The kidneys have multiple roles in systemic glucose metabolism, such as glucose reabsorption, gluconeogenesis, and insulin degradation. Therefore, putative renal hyperfunction might contribute to the development of T2D. The present study characterized rats from a strain of novel type 2 diabetes model with enlarged kidneys (DEK). Their kidneys have increased parenchyma (nephrons and tubules), and uninephrectomy immediately after the onset inhibited the development of T2D for a significant period in DEK rats. These results highlight the contribution of kidney to the development of T2D, and indicate that the kidneys are therapeutic targets to prevent T2D.

**Abstract:**

The kidneys participate in the regulation of systemic glucose metabolism via gluconeogenesis, insulin degradation, and the tubular reabsorption of glucose. The present study characterized rats from a strain of a novel type 2 diabetes model with enlarged kidneys (DEK). Histological and biochemical analyses of DEK rats were performed to assess the relationships between their kidneys and hyperglycemia. The kidney weight of diabetic DEK (DEK-DM) gradually increased over time from the onset of diabetes, with the glomerular number being higher in DEK-DM than in normal DEK (DEK-cont). A positive correlation between blood glucose level and kidney weight was observed in DEK-DM. The similar glomerular size and single glomerular creatinine clearance in DEK-cont and DEK-DM indicated that glomerular hypertrophy and hyperfiltration were not involved in the renal enlargement. Uninephrectomy (1/2Nx) in DEK-DM resulted in a reduction in blood glucose level at 7–28 post-operation days, with this concentration remaining lower than in Sham group until 84 days post-operation. 1/2Nx also improved systemic conditions, including reduced body weight gain, polyuria, polydipsia, and hyperphagia. Plasma concentrations of Na, total cholesterol, albumin, and total protein were higher, and urinary excretion of glucose, urea nitrogen, and proteins were lower, in the 1/2Nx than in the Sham group. Remnant kidney weight was two-fold higher in the 1/2Nx than in the Sham group 84 days later. In addition, 1/2Nx resulted in renal tubular dilatation but not in the progression of fibrosis or glomerular lesions. Taken together, these findings indicate that enlarged kidneys were associated with the onset of diabetes and with the resistance to diabetic nephropathy in DEK-DM.

## 1. Introduction

The worldwide prevalence of diabetes mellitus (DM) in 2020 has been estimated at 463 million patients. DM is largely classified into types 1 and 2 diabetes (T1D and T2D). T1D, which affects about 10% of patients with DM, is characterized by insulin deficiency due to the degradation of islet β cells; whereas T2D, which affects about 90% of these patients, is characterized by a combination of insulin resistance with insulin deficiency [1,2]. Pathologically, an underlying hyperglycemic condition in patients who develop T2D is thought to induce β cell dysfunction, leading to impaired glucose tolerance and DM. Although hyperglycemia condition and β cell deficiency are complex abnormalities involved in numerous metabolic pathways and organ systems, the complete pathogenic mechanism inducing T2D remains unknown [2,3].

Drugs currently used in the treatment of DM vary in their mechanisms of action, including the stimulation of insulin secretion, the suppression of hepatic gluconeogenesis, the inhibition of intestinal glucose absorption, and the reduction of insulin resistance in liver and muscle. Inhibitors of sodium-glucose cotransporter 2 (SGLT2) are a novel class of anti-diabetic drugs with a different mechanism of action, reducing blood glucose concentrations by inhibiting glucose reabsorption in renal tubules [3,4]. SGLT2 inhibitors have been shown to have a low risk of hypoglycemia in diabetic patients and to reduce the incidence of obesity, cardiovascular diseases, and diabetic nephropathy [5,6,7]. Although studies have assessed the role of the kidneys in glucose homeostasis, less is known about the kidneys as a therapeutic target for diabetes [4]. However, the success of SGLT2 inhibitors in the treatment of DM has highlighted the kidneys as a target in DM treatment [3,4].

The kidneys, which have high energy demands, take up glucose from the blood, while contributing to higher blood glucose levels via gluconeogenesis, insulin degradation, and tubular reabsorption of glucose [3,4,5,8,9]. In a fasting state, the kidneys release lower amounts of endogenous glucose into the circulation than the liver. This relationship, however, is reversed after ingestion of food, due to the suppression of gluconeogenesis and the promotion of glycogenesis in the liver [8,10,11]. Thus, the kidneys likely release glucose into the circulation, even when blood glucose levels are high. The amount of glucose released from the liver was found to be only ~30% higher in patients with DM than in non-diabetic individuals, whereas glucose release from the kidneys was three-fold in patients with DM [12]. In addition, individuals with prediabetes and early stages of DM have enlarged kidneys and increased gluconeogenesis and glucose reabsorption, along with the growth of renal tubules [13,14,15].

Despite the involvement of the kidneys in various aspects of glucose metabolism, less is known about the involvement of the kidneys in the onset and aggravation of DM [4]. The total number of nephrons in human kidneys has been reported to vary over a 10-fold range [16], suggesting that the renal parenchyma related to glucose metabolism also differs congenitally among individuals. We have established a novel non-obese T2D rat strain, DEK, characterized by the loss of pancreatic β cells and enlarged kidneys, but with nearly normal renal function [17]. The present study investigated whether the kidneys could accelerate diabetes in DEK rats.

## 2. Materials and Methods

### 2.1. Animals

The present study included 68 male DEK rats aged 15 to 45 weeks [17]. Blood was obtained from the tail veins of non-fasting rats, and blood glucose levels were measured with GLUCOCARD My DIA (Arkray Inc., Kyoto, Japan). Rats with blood glucose concentrations < 300 mg/dL were classified into the DEK-control (cont) group (*n* = 21) and those with blood glucose concentrations ≥ 300 mg/dL into the DEK-diabetes mellitus (DM) group (*n* = 47), as described [17]. All rats were maintained in a clean conventional animal room under a 14:10 h light:dark cycle, with room temperature and humidity maintained at 20 ± 2 °C and 50% ± 10% respectively [17], and ad libitum access to water and a standard diet (CR-LPF, Oriental Yeast Co., Ltd., Tokyo, Japan). All studies in animals were approved by the Animal Care and Use Committee of Nippon Veterinary and Life Science University and were performed in accordance with Guidelines of the Animal Care and Use Committee of Nippon Veterinary and Life Science University.

### 2.2. Determination of Glomerular Number (GN)

Thirty-week-old DEK rats were kept in metabolic cages, and 24 h urine samples were collected. These rats were subsequently anesthetized with pentobarbital, and blood samples were collected from the jugular vein. After injecting India ink and euthanizing the animals, their kidneys were removed and weighed with an analytical balance. Glomeruli were counted by HCl-maceration methods, as described [18,19]. The GN and kidney weight of the individual rat were presented as the average of two kidneys on both sides.

### 2.3. Measurement of Glomerular Size

Glomerular size was measured as previously reported [19]. Briefly, more than ten glomeruli from each individual kidney were photographed under a light microscope (OLYMPUS BX50; Shinjuku-ku, Japan). The glomerular projective area was measured by Image J software.

### 2.4. Nephrectomy

Rats aged 15–21 weeks in the DEK-DM group were randomly classified into two subgroups, placed under inhalation isoflurane anesthesia, and subjected to uninephrectomy (1/2Nx) or a sham operation (Sham), as described [20]. In the 1/2Nx subgroup, the left kidney was surgically removed through a small flank incision, whereas, in the Sham subgroup, the left kidney was exposed but not removed. Body weight and glucose concentration in tail vein blood were measured weekly. The rats were placed in metabolic cages at 28 and 84 days after the surgical operation for collection of 24 h urine samples. After 28 days, the rats were placed under inhalation isoflurane anesthesia and blood samples were collected from the jugular vein. After 84 days, the rats were euthanized with overdoses of pentobarbital, blood samples were collected from the caudal vena cava, and the kidneys were collected for histological analysis. Twenty-eight-day kidney samples were also obtained from other rats for histological examination. Urine volume, water intake, and food intake for 24 h were measured as described previously [17,19].

### 2.5. Immunofluorescence and Pathological Analysis of Kidney

Kidneys were weighed and fixed with 4% paraformaldehyde in phosphate-buffered saline (PBS). The fixed kidneys were rinsed with PBS, dehydrated, embedded in paraffin, and sectioned into 2–4 μm thick sections for hematoxylin and eosin (H&E), periodic acid-Schiff (PAS), and Masson’s trichrome (MT) staining [17]. Renal tubule lumen areas were measured using Image J software. The kidney sections were rehydrated, autoclaved at 105 °C for 5 min, blocked by incubation with 10% BSA in PBS for 30 min, and incubated with 0.3% hydrogen peroxide in PBS to inactivate any endogenous peroxidases. The sections were subsequently incubated with mouse anti-proliferating cell nuclear antigen (PCNA) monoclonal antibody (1:100, Santa Cruz Biotechnology Inc., Santa Cruz, CA, USA) overnight at 4 °C, rinsed with PBS, and incubated with horseradish peroxidase (HRP)-conjugated donkey anti-mouse antibody (1:1000, Santa Cruz Biotechnology Inc.) for one hour at room temperature. Immunoreactivity was visualized with 3,3′-diaminobenzidine (DAB) substrate (BD Pharmingen, San Diego, CA, USA). The number of PCNA-positive cells was estimated as an average of counts in 10 pictures.

### 2.6. Biochemical Analysis

Plasma concentrations of total cholesterol (Tcho), urea nitrogen (UN), Na-K-Cl, albumin (Alb), and total protein (TP) were measured with DRI-CHEM3500V (FUJIFILM, Tokyo, Japan) [17,21], and plasma Cre concentrations were measured by FUJIFILM VET Systems Co., Ltd. (Tokyo, Japan). Urinary concentrations of creatinine (Cre), UN, electrolytes (Na, K, and Cl), and glucose (Glu) were measured with DRI-CHEM3500V (FUJIFILM), and urinary protein concentrations were measured with the Protein Assay Rapid Kit Wako II (FUJIFILM Wako Pure Chemical Corporation, Tokyo, Japan). Total 24 h urinary excretions of Cre, UN, electrolytes, protein, and glucose were calculated from measured concentrations and volumes.

Clearance (C) of each substance (x) was calculated using the following formula:

Cx (mL/min/kg) = [urine concentration (mg/dL or mEq/L) × urine volume (mL/min)]/plasma concentration (mg/dL or mEq/L)/body weight (kg).

Fractional excretion (FE) of each substance (x) was calculated using the formula:

FEx (%) = Cx (mL/min/kg)/Ccr (mL/min/kg) × 100.

Single nephron creatinine clearance (SNCcre) was calculated by dividing Ccre by total GN.

### 2.7. Oral Glucose Tolerance Test (OGTT)

The oral glucose tolerance test (OGTT) was performed as described [17]. Briefly, after a 16 h fast, rats were orally administered 2 g/kg body weight (BW) of glucose, and glucose levels were measured in tail vein blood at 0, 30, 60, 120, 180, and 240 min. Blood collected from the jugular vein under isoflurane anesthesia at 0, 30, and 60 min after glucose loading was mixed with heparin and centrifuged for 15 min at 4 °C, and plasma insulin concentrations were measured using an LBIS Rat Insulin ELISA Kit (RTU) (Shibayagi, Gunma, Japan).

### 2.8. Statistical Analysis

Results were presented as means ± standard error (SE), and statistical significance was determined using the two-tailed Student’s *t*-test. For multiple comparison, one-way ANOVA with Bonferroni correction was performed. Correlation coefficients between pairs of parameters (kidney weight, GN, and blood glucose concentration) were calculated by Pearson correlation analysis, with the *p*-values of correlation coefficients calculated by the *t*-distribution function. All statistical analyses were performed using Excel 2017 for Mac (MicroSoft Corp., Redmond, WA, USA), with statistical significance defined as *p* < 0.05.

## 3. Results

### 3.1. Characterization of Enlarged Kidneys in DEK-DM Rats

**Figure 1:**
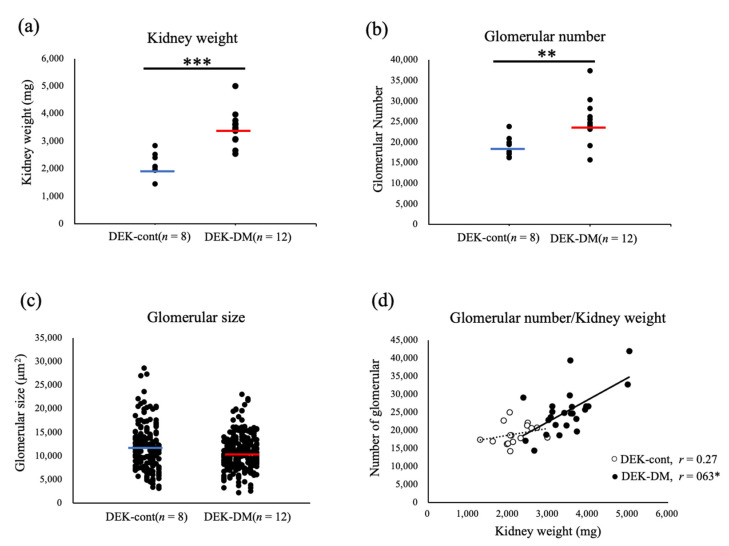
Analysis of glomerular number and size. Scattergrams showing mean (**a**) kidney weight and (**b**) glomerular number (GN) of individual rats. ** *p* < 0.01, *** *p* < 0.001 for between-group differences. The blue and red horizontal bars indicate averages for DEK-cont and DEK-DM rats, respectively. (**c**) Scattergram showing raw glomerular sizes (μm^2^) of a total of 400 glomeruli from both kidneys of DEK-cont and DEK-DM rats. (**d**) Bilateral GN and kidney weights from individual rats plotted as white circles in DEK-cont rats and black circles in DEK-DM rats, with correlation coefficient shown as r. * *p* < 0.05 for correlation.

DEK-DM rats were found to be hyperglycemic at age 15–21 weeks, whereas DM-cont rats were not. To characterize the differences between their kidneys, DEK-cont and DEK-DM rats were sacrificed at age 30 weeks, and their kidneys were weighed, the numbers of GN counted, and glomerular size measured. DEK-DM rats had significantly heavier kidneys (3463.5 ± 132.3 mg vs. 2161.0 ± 105.0 mg, *p* < 0.001) (Figure 1a) and significantly higher GN than DEK-cont rats (25,240.0 ± 1554.0 vs. 18,970.0 ± 916.8, *p* < 0.01) (Figure 1b). Although diabetes is generally accompanied by glomerular hypertrophy, glomerular size was similar in DEK-DM and DEK-cont rats (10,971.8 ± 236.0 vs. 11,870.6 ± 358.5 µm^2^, *p* > 0.05) (Figure 1c), suggesting that enlarged kidneys of DEK-DM rats are not accompanied by glomerular hypertrophy. This was supported by a significant positive correlation between kidney weight and GN in DEK-DM rats, indicating that enlarged kidneys were associated with increased GN (Figure 1d). In addition, SNCcre (0.34 ± 0.06 vs. 0.26 ± 0.08 µL/min/kg) tended to be higher in DEK-DM than in DEK-cont rats, but the differences were not statistically significant. These results indicate that enlarged kidneys were characterized by an increased number of nephrons without glomerular hypertrophy or hyperfiltration.

### 3.2. Relationship between Kidney Weight and Hyperglycemia

**Figure 2:**
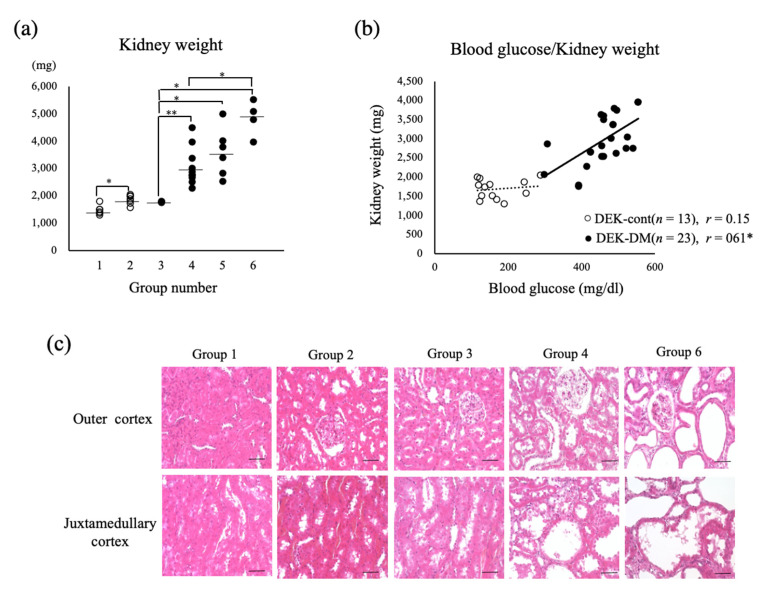
Association between kidney weight and blood glucose. (**a**) Scattergram showing mean kidney weights (mg) in individual DEK-cont (white circles) and DEK-DM (Block circles) rats classified into six age groups. Group 1: DEK-cont rats aged 15 weeks (*n* = 6); Group 2: DEK-cont rats aged 30 weeks (*n* = 7); Group 3: DEK-DM rats euthanized just after diabetes onset at age 15~22 weeks (*n* = 3); Group 4: DEK-DM rats with late-onset (>18 weeks) DM euthanized at age 30 weeks (*n* = 10); Group 5: DEK-DM rats with early-onset (~15 weeks) DM euthanized at age 30 weeks (*n* = 7); Group 6: DEK-DM aged 42~45 weeks after having DM for 21–27 weeks (*n* = 4). The horizontal bars represent averages. * *p* < 0.05, ** *p* < 0.01. (**b**) Relationship of blood glucose concentration (X-axis) and kidney weight (Y-axis) in individual DEK-cont (white circles) and DEK-DM (black circles) rats aged 15–30 weeks, with the correlation coefficient shown as r. * *p* < 0.05 for correlation. (**c**) Histology of outer and juxtamedullary cortices of representative kidneys in each group categorized in (**a**). H & E staining (scale bar = 100 µm).

**Figure 3:**
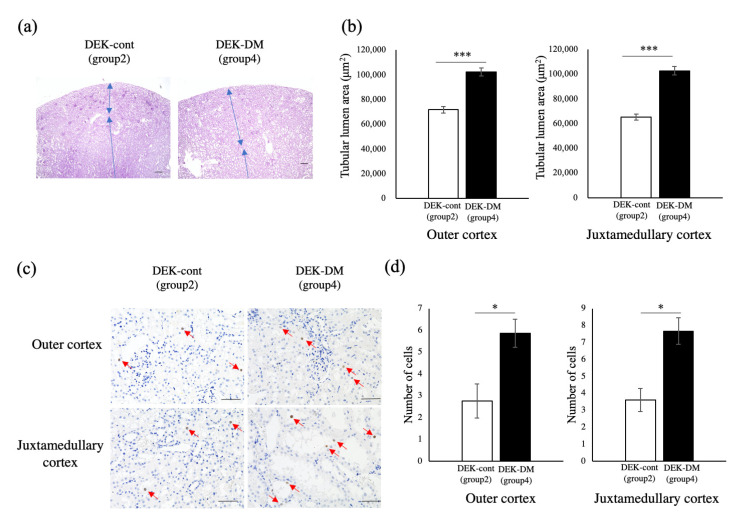
Histological analysis of kidney. Detailed analysis of renal histology of DEK-cont (Group 2) and DEK-DM (Group 4) rats, as categorized in Figure 2a. (**a**) Cortical areas were occupied by dilated renal tubules in DEK-DM, resulting in the number of glomerular sections appearing to be lower in DEK-DM than in DEK-cont rats. Scale bar = 200 μm. Histologically identical areas are indicated by double-ended arrows. (**b**) Areas of tubular lumen were quantitatively compared. *** *p* < 0.001 for between-group differences. (**c**) Immunohistochemistry of PCNA in outer and juxtamedullary cortices. Typical PCNA-positive cells are indicated by red arrows. Scale bar = 50 μm. (**d**) Numbers of PCNA-positive cells in (**c**). Group 2: DEK-cont rats aged 30 weeks (*n* = 3). Group 4: DEK-DM rats with late-onset (>18 weeks) DM euthanized at age 30 weeks (*n* = 3). * *p* < 0.05 for between-group differences.

To evaluate the relationship between kidney enlargement and diabetic condition, renal weight was compared in six groups of rats categorized by age at onset of DM: Group 1, DEK-cont rats aged 15 weeks; Group 2, DEK-cont rats aged 30 weeks; Group 3, DEK-DM rats euthanized just after diabetes onset at age 15~22 weeks; Group 4, DEK-DM rats with late-onset (>18 weeks) DM euthanized at age 30 weeks; Group 5, DEK-DM rats with early-onset (~15 weeks) DM euthanized at age 30 weeks; Group 6, DEK-DM aged 42~45 weeks after having DM for 21–27 weeks (Figure 2a). Average kidney weights in groups 1 and 2 were 1483.8 ± 70.4 mg and 1844.0 ± 63.5 mg respectively, indicating that the normal kidney weight gain was 1.2-fold for 15 weeks. The average kidney weight in group 3 just after onset of DM was 1773.4 ± 9.3 mg, similar to non-diabetic kidney weight in groups 1 and 2, and indicating that the kidney weight in DEK-DM rats was normal at the onset of diabetes. Average kidney weights in groups 4 and 5 were 2966.6 ± 249.2 mg and 3594.8 ± 364.0 mg respectively, or 1.7- and 2.0-fold higher respectively, than in group 3, indicating that a longer period of diabetes was accompanied by increased kidney weight. The average kidney weight in group 6, the group with the longest duration of diabetes, was 4844.0 ± 325.7 mg. Consistent with these observations, we found significant correlation between kidney weight and blood glucose level in DEK-DM but not in DEK-cont rats (Figure 2b). Histological examination showed that the area of tubular lumen gradually increased from group 1 to group 6, with severe dilation of renal tubules observed in groups 4 to 6 (Figure 2c and Figure 3b). To assess the cause of these increases in kidney weight, we compared renal sections in DEK-cont (group 2) and DEK-DM (group 4) rats. The density of glomeruli in DEK-DM rats was lower and was associated with the increase in cortical area, mostly occupied by significantly dilated tubules (Figure 3a,b). The numbers of PCNA-positive cells in both the outer and juxtamedullary cortices were increased in DEK-DM rats (Figure 3c,d), indicating a greater proliferation of renal tubular cells in DEK-DM than in DEK-cont rats. These findings suggested that higher kidney weight in DEK-DM rats was accompanied by the increase in dilated renal tubules, a condition associated with the severity of diabetes.

### 3.3. Effect of Uninephrectomy (1/2Nx) on General Conditions Related to Diabetes

The above findings indicated that enlarged kidneys in DEK-DM were involved in the increases of renal parenchyma, including increases of nephrons and tubules. Since kidneys are associated with glucose homeostasis via gluconeogenesis, insulin clearance, and glucose reabsorption, we hypothesized that the increased renal function associated with increased parenchyma may contribute to the induction of hyperglycemia in DEK-DM rats. To verify this hypothesis, DEK-DM rats were subjected to 1/2Nx, and the effect of the reduction in renal parenchyma was evaluated. Since data from rats of normal strains are informative to evaluate some parameters, 1/2Nx was also performed on Wistar male rats, and their data were included in the supporting information (Supplementary Tables S1–S4).

**Figure 4:**
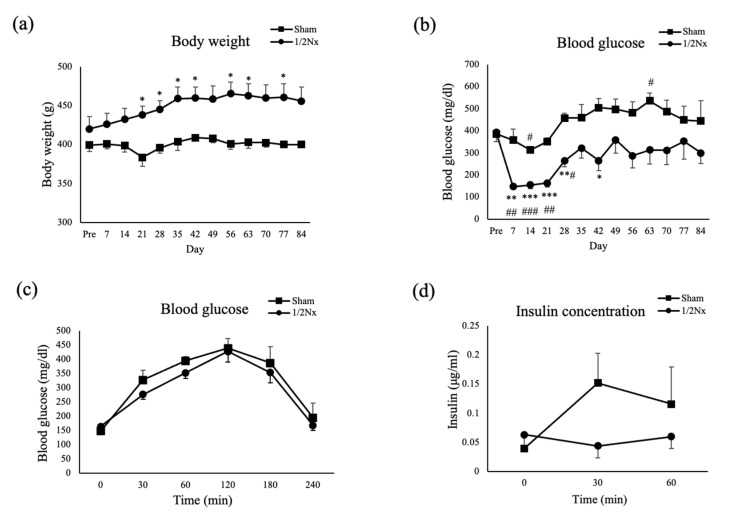
The changes of body weight and blood glucose after surgery and oral glucose tolerance test at 28 days after surgery. (**a**,**b**) Postoperative changes in (**a**) body weights and (**b**) blood glucose concentrations of Sham operated (*n* = 3) and 1/2Nx (*n* = 6) rats. * *p* < 0.05, ** *p* < 0.01, *** *p* < 0.001 between the two groups. # *p* < 0.05, ## *p* < 0.01, ### *p* < 0.001 compared with preoperative value in the same group. (**c**,**d**) OGTT results 28 days after surgery in the Sham operated (*n* = 3; closed squares) and 1/2Nx (*n* = 4; closed rhombi) rats. (**c**) Blood glucose level after glucose loading. (**d**) Plasma insulin level after glucose loading.

We found that body weight gain was inhibited in Sham operated rats, as shown in non-treated DEK-DM rats [17,22]. In contrast, body weight gradually increased with age in 1/2Nx rats, with body weight after 21 days being significantly higher in 1/2Nx than in Sham operated rats (Figure 4a).

Moreover, blood glucose concentrations also differed after the surgical operation. In the Sham group, blood glucose level decreased at 14 days, but subsequently increased, to over 450 mg/dL. In contrast, blood glucose levels in the 1/2Nx group decreased markedly to a normal level (<200 mg/dL) on days 7–28 after the surgical operation, being subsequently maintained at an almost constant level near the preoperative level (about 300 mg/dL), until day 84. Thus, blood glucose levels were significantly lower in 1/2Nx than in Sham operated rats at 7, 14, 21, 28, and 42 days after surgery (Figure 4b).

To evaluate insulin secretion in Sham operated and 1/2Nx rats during the period when blood glucose levels were low, these rats were subjected to OGTTs 28 days after surgery. Both Sham operated and 1/2Nx rats showed similar changes in response to glucose loading, with blood glucose concentrations maintained at >300 mg/dL from 60 to 180 min and peaking at over 400 mg/dL at 120 min (Figure 4c). Consistent with these findings, there was no significant secretion of insulin in both groups of rats (Figure 4d).

The absolute weight of the remnant kidney of 1/2Nx rats was significantly higher than that of Sham operated rats at 28 days, and both the absolute and relative weights were greater in 1/2Nx than in Sham operated rats at 84 days (Table 1). The weight of the remnant kidney in 1/2Nx rats at 84 days was more than double that on the day of operation and close to double that of Sham operated rats. This compensatory response in remnant kidney was markedly large compared to data from Wistar rats (Supplementary Table S1).

In addition to polyuria and polydipsia, DEK-DM rats show hyperphagia [17]. We therefore measured 24 h food intake, water intake, and urinary volume in 1/2Nx and Sham operated rats at 28 and 84 days. At 28 days, these parameters were significantly lower in 1/2Nx than in Sham operated rats (Table 2). This tendency was also observed at 84 days, although the two groups did not differ significantly in water intake and urinary volume (Table 2). No effect of 1/2Nx on these parameters was observed in Wistar rats (Supplementary Table S2).

### 3.4. Effect of Uninephrectomy on Biochemical Parameters

To evaluate metabolic status and renal function, biochemical parameters were measured in plasma and urine. A previous comparison of DEK-DM and DEK-cont rats showed that plasma Cre, Tcho, electrolytes, Alb, and TP concentrations were lower, while excretion of Glu, urea nitrogen, electrolytes, and protein into urine were higher, in DEK-DM than in DEK-cont rats [17,22]. This condition was partially reversed by 1/2Nx (Table 3 and Table 4).

Plasma levels of Tcho and Cre were higher in 1/2Nx than in Sham operated rats at 28 and 84 days (Table 3), although plasma Cre levels were lower than the level suspected in renal dysfunction. Plasma Na concentrations were higher in 1/2Nx than in Sham operated rats at 28 days. Plasma Alb and TP concentrations were significantly higher in 1/2Nx than in Sham operated rats at 84 days (Table 3), indicating an improvement in hypoproteinemia.

Urinary UN excretion, which was enhanced in Sham operated rats, was significantly lower in 1/2Nx rats at 84 days. However, there was no between-group difference in BUN, indicating the improvement of catabolic conditions. Urinary excretion of Cl was significantly lower in 1/2Nx than in Sham operated rats at 84 days. Similarly, urinary excretions of other electrolytes were lower in 1/2Nx rats, although the between-group differences were not statistically significant. High-level urinary excretions of protein and glucose in Sham operated rats were also significantly alleviated in 1/2Nx at 28 days, with this tendency also observed at 84 days (Table 4).

Consistent with the 2-fold reduction in the number of nephrons in 1/2Nx rats, Ccre was also reduced in 1/2Nx rats, with the between-group difference being significant at 84 days (Table 5). Based on Wistar rat data (Supplementary Table S3), however, this Ccre in 1/2Nx rats was still at a normal level. In agreement with the extremely low level of urinary glucose excretion in 1/2Nx rats at 28 days (Table 4), Cglu was significantly lower in 1/2Nx than in Sham operated rats at 28 days (Table 5). Although the difference was not statistically significant, urinary excretion of Na was lower in 1/2Nx than in Sham operated rats (Table 4). This condition was accompanied by a decrease in FEna of 1/2Nx rats (Table 5), indicating increased absorption by renal tubules at 28 days.

### 3.5. Histology of Nephrectomized Kidneys

**Figure 5:**
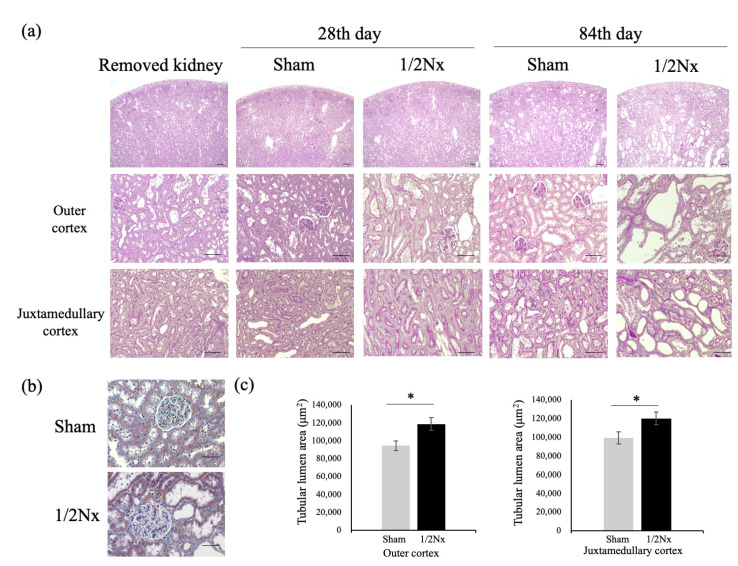
Histological analysis of kidney after surgery. Histological analysis of renal tissues from Sham operated and 1/2Nx rats on the day of operation (removed kidney) and 28 and 84 days after surgery. (**a**) PAS staining showing renal tubular dilation in cortices. The top panels show images at low magnification (scale bar = 200 μm), whereas the middle and bottom panels show images at high magnification of the outer and juxtamedullary cortices, respectively (scale bar = 50 μm). (**b**) MT staining showing glomerulus structures 84 days after surgery (scale bar = 20 μm). Mild fibrosis was observed in 1/2Nx glomeruli. (**c**) Areas of tubular lumen in Sham operated and 1/2Nx rats 84 days after the operation. MT staining; scale bar = 50 μm. *n* = 3 with at least three randomly selected sections in each area analyzed in each group. * *p* < 0.05 between-group differences.

Figure 5 shows the histology of kidneys in the 1/2Nx and Sham operated groups before and 28 and 84 days after surgery. We previously reported that histologically, DEK-DM rats are characterized by dilated renal tubular rather than glomerular lesions [17]. Compared with preoperatively examined kidneys, renal tubular lesions, such as tubular dilation, loss of brush borders, and epithelial flattening, appeared to be more severe in 1/2Nx than in Sham operated rats at 28 days, and more severe at 84 than at 28 days (Figure 5a). The areas of tubular lumen in outer and juxtamedullary cortices were significantly larger in 1/2Nx than in Sham operated rats at 84 days (Figure 5c). In contrast, glomerular and interstitial tissues remained almost normal, with no clear evidence of fibrosis in both the Sham operated and 1/2Nx rats. We observed minimal increases in collagen fibers, as shown by aniline dyeing, around the glomeruli in 1/2Nx rats (Figure 5b).

**Figure 6:**
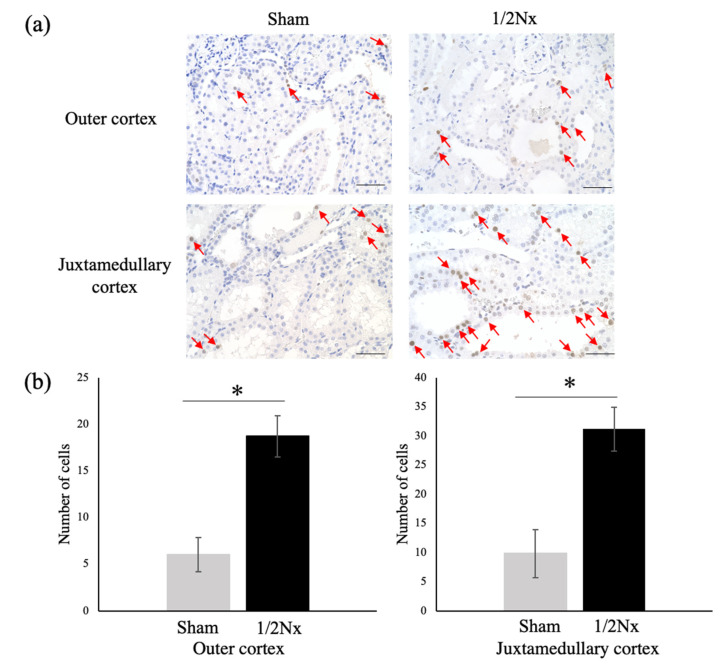
Immunohistochemical analysis of proliferation activity in the renal tubules in Sham operated and 1/2Nx DEK rats 84 days after surgery. (**a**) Immunohistochemistry of PCNA in the outer and juxtamedullary cortices. Typical PCNA-positive cells are indicated by red arrows. Scale bar = 50 μm. (**b**) Numbers of PCNA-positive cells per unit area. *n* = 3 with at least three randomly selected sections in each area analyzed in each group. * *p* < 0.05 between the two groups.

To characterize rapid increases in the weights of remnant kidneys after 1/2Nx, we analyzed the proliferation activity of renal tubules using PCNA staining. We found that the number of PCNA-positive cells was higher in remnant kidneys of 1/2Nx than of Sham operated rats (Figure 6a,b). The numbers of PCNA-positive cells in 1/2Nx and Sham operated rats is more than three times the value of each in Wistar rats (Supplementary Table S4).

## 4. Discussion

Renal size increases during early stages of diabetes and is usually associated with glomerular hyperfiltration and hypertrophy [23,24,25]. In experimental animals, this condition is markedly influenced by the metabolic state. For example, kidney weight increased in moderate hyperglycemia, whereas renal enlargement was inhibited in severe hyperglycemia (blood glucose > 450 mg/dL) [15]. The present study found that kidney weight in DEK-DM rats gradually increased with age after the onset of diabetes. The positive correlation between blood glucose level and kidney weight in DEK-DM rats was found in hyperglycemic condition from 300 to 600 mg/dL of blood glucose, unlike the previous study [15]. Furthermore, the similar glomerular sizes and SNCcre in DEK-cont and DEK-DM rats indicated that glomerular hypertrophy and hyperfiltration were not involved in renal enlargement in DEK-DM. Interestingly, we found that the number of glomeruli was significantly higher and correlated positively with kidney weight in DEK-DM rats, indicating that enlarged kidneys were involved in the increase in nephron number. In rats, nephrogenesis ends soon after birth, with nephron precursor stem cells being absent from adult kidneys. Thus, the increase in nephron number could not have been caused by the elevation of blood glucose levels in DEK-DM rats. These findings indicate that the increase in nephron endowment occurred prior to the onset of diabetes and was a prerequisite for the development of enlarged kidneys and possibly diabetes. Renal tubules correspond to 90% of the parenchyma in kidneys. Histological examination of the kidneys in DEK-DM rats showed that all renal areas, including the outer and juxtamedullary cortices (Figure 3a) and the outer and inner medulla, increased in size [17]. This alteration was accompanied by a reduced density of glomeruli in cortical sections, suggesting increased mass of renal tubules with dilation in the enlarged kidneys of DEK-DM rats. Therefore, the increase in renal parenchyma involved in the increases of both nephrons and renal tubules is likely a prerequisite for the development of diabetes.

Nephron endowment is a critical factor directly influencing renal function. The renal dysfunction occurs in rats with reduced nephron number resulted from bilateral renal hypoplasia and unilateral renal agenesis [18,21,26]. The total number of nephrons in normal human kidneys has been reported to vary over a 10-fold range, with subjects having a low nephron number tending to have glomerular hyperfiltration and hypertrophy, as well as glomerulosclerosis [16]. In contrast, high nephron number is thought to reduce the risks of glomerular filtration rate (GFR) deficiency and glomerulosclerosis by protecting against glomerular volume heterogeneity, including hypertrophy [27]. Therefore, a higher number of glomeruli and glomeruli of homogeneous size in DEK-DM rats may indicate congenital resistance to diabetic nephropathy. In general, a high reserve capacity of organ will not appear under normal conditions, since the function status basically depends on its demand. When the supply of feeding vessels and working vessels is common, an increase in the functional units of tissue may be accompanied by hyperfunction. The present study found that SNCcre, as an indicator of single nephron GFR, did not decrease in hyperglycemic DEK-DM rats, even those with a high nephron number. Increased total GFR shortens the half-life of circulating insulin [9,28]. In addition, glucose reabsorption and gluconeogenesis have been reported to increase under prediabetic and diabetic conditions [5,29]. Furthermore, renal tubules are the main tissues responsible for insulin degradation, glucose reabsorption, and gluconeogenesis in the kidneys [28,29]. Therefore, high functioning, substantial kidneys with increased nephron number and renal tubules in DEK-DM rats may be a precondition for the development and deterioration of diabetes. DEK-DM rats are non-obese and show noninflammatory reductions in islet β cells [17]. Enlarged kidneys may contribute to potential insulin resistance by inducing a potentially hyperglycemic condition. Although little is known about the ability of highly functional kidneys to induce diabetes in humans, reduced renal dysfunction increases the risk for hypoglycemia during drug therapy for diabetes [30], with an onset of post-transplant DM observed in 15~40% of kidney recipients [31]. These findings suggest that alterations in renal function are associated with the onset and progression of diabetes.

To obtain direct evidence showing that increased nephron mass contributes to the development of diabetes, we subjected DEK-DM rats to 1/2Nx immediately after the onset of diabetes. Generally, 1/2Nx in a diabetic animal model accelerates the development of diabetic nephropathy because of chronic hyperglycemia and the compensatory load on the residual kidney [20,32]. In contrast, all DEK-DM rats that underwent 1/2Nx showed a reduction in blood glucose level 28 days after the operation, with glucose concentration being lower than in Sham operated rats for 84 days. In addition, 1/2Nx improved systemic conditions in these animals, reversing the suppression of body weight gain, polyuria, polydipsia, and hyperphagia. These sustained improvements suggest that 1/2Nx could inhibit the progression of glucose intolerance in DEK-DM rats. SGLT2 inhibitors normalized blood glucose by inhibiting glucose reabsorption in renal tubules [4,5,6,7]. In addition, SGLT2 inhibitors may inhibit renal glycogenesis under prediabetic conditions [29]. Long-term administration of the SGLT2 inhibitor, empagliflozin, reduced blood glucose level and improved systemic conditions in DEK-DM rats [22]. In addition, 1/2Nx was found to increase insulin sensitivity, whereas uremic conditions reduce this sensitivity, in Wistar rats, suggesting that reduced gluconeogenesis capacity resulted from the partial improvement in insulin resistance induced by 1/2Nx [33]. In our study, the lack of insulin secretory response, together with glucose intolerance, in both 1/2Nx and Sham operated rats suggested that improvements induced by 1/2Nx may be independent from insulin signaling in DEK-DM rats. Therefore, the overall improvements caused by the SGLT2 inhibitor and 1/2Nx are thought to be due to reductions in renal function associated with elevated blood glucose concentrations.

Removal of one kidney from DEK-DM rats increased the weight of the remaining kidney, with this kidney in 1/2Nx rats being two-fold heavier than the kidneys in Sham operated rats at 84 days. This finding was consistent with results showing that the combination of diabetes and 1/2Nx accelerated renal growth [15]. Since blood glucose concentrations in these 1/2Nx rats were almost normal, hyperglycemia was not considered a major cause of renal enlargement. Similarly, our previous study revealed that the normalization of blood glucose level by the administration of empagliflozin did not prevent renal enlargement in DEK-DM rats [22]. Therefore, the intrinsic growth ability of these kidneys may be enhanced by 1/2Nx. Following 1/2Nx, a compensatory mechanism acts on the remnant kidney, enhancing both renal function and size [34,35]. Since the enlarged kidneys with an increased number of nephrons in DEK-DM rats were highly functional, plasma Cre concentration and total Ccre were still within normal ranges 84 days after 1/2Nx, despite Cre being higher and total Ccre being lower in DEK-DM than in Sham operated rats. Taken together, these findings indicate that the excretion function of the single remnant kidney in DEK-DM was not reduced compared to that of two kidneys in non-operated normal rats.

1/2Nx was also found to increase the plasma concentrations of Tcho, Na, Alb, and TP and to reduce the urinary excretions of glucose, UN, and protein in DEK-DM rats. Interestingly, these alterations were also observed in DEK-DM rats treated with empagliflozin [22], suggesting that these alterations may have been due to improvements in negative energy balance resulting from easing of diabetic conditions. That is, the conversion from a hypercatabolic to an anabolic state may reduce the production of urea nitrogen and enhance the production in the liver of plasma proteins, including albumin. Increased albumin production may, in turn, enhance the production of Tcho [36]. The administration of empagliflozin may also increase the tubular reabsorption of electrolytes. Although this was unclear in 1/2Nx rats, water reabsorption was enhanced, an alteration that may have been due to improvements in osmotic diuresis caused by the reduced filtration of glucose, with the latter resulting from normalization of blood glucose levels.

1/2Nx in other animal models of diabetes was found to increase glomerular filtration rate (GFR) and proteinuria, with progression to diabetic nephropathy accompanied by the expansion of the mesangium region in glomeruli [20,32,37]. In contrast, 1/2Nx of DEK-DM rats resulted in normal renal function in the remaining, highly enlarged kidneys. In addition, the 1/2Nx rats showed renal tubular dilatation 84 days later, but there was no evidence of progression of marked fibrosis or glomerular lesions. An increase in the number of PCNA-positive cells has been shown to correlate with renal tubular regeneration and cystic formation [38,39]. Therefore, the increased number of PCNA-positive epithelial cells in DEK-DM is considered to reflect the increased number of renal tubules and the progression of tubular dilation. Therefore, the increase in PCNA-positive cells in 1/2Nx DEK-DM rats further indicates enhancement of the proliferation of renal tubules. This condition might compensate for the degradation of tubular and glomerular cells. The blood glucose level that dropped rapidly after 1/2Nx was increased 28 days after 1/2Nx, indicating that the renal function contributing to hyperglycemia might be rapidly enhanced in remnant kidney along with a rapid increase of parenchyma.

Taken together, these findings indicate that enlarged kidneys in DEK-DM rats were associated with the onset of diabetes and are resistant to diabetic nephropathy. Larger renal parenchyma, including increases in the numbers of nephrons and renal tubules, could be sufficiently functional to contribute to hyperglycemia, a condition that may be suppressed by deletion of renal parenchyma, as in 1/2Nx. These results suggest that populations with high nephron number may be more susceptible to diabetes induced by environmental and genetic factors, but may be more resistant to kidney diseases such as diabetic nephropathy. Since no diabetic model with a substantial increase in nephron number has been reported, DEK rats are a unique animal model for studying the relationship between renal function and diabetes.

## 5. Conclusions

The enlarged kidney of DEK rats is characterized by increased parenchyma (nephrons and tubules) and contributes to the onset and progression of diabetes.

## Figures and Tables

**Table 1:** Kidney weights before surgery (Pre) and 28 and 84 days after surgery in Sham operated and 1/2Nx DEK rats.

	Kidney Weight (g)			Relative Kidney Weight (g/kg)		
	Pre/L Side	28 Days/R Side	84 Days/R Side	Pre/L Side	28 Days/R Side	84 Days/R Side
Sham	-	2.1 ± 0.1	2.9 ± 0.2	-	4.7 ± 0.2	7.6 ± 0.4
1/2Nx	2.1 ± 0.1	2.6 ± 0.1 ***	5.5 ± 0.4 ***	5.0 ± 0.2	6.0 ± 0.5	12.1 ± 0.7 **

The average kidney weights of left (L) kidneys removed from 1/2Nx DEK rats (Pre, *n* = 10), right (R) kidneys of Sham operated (*n* = 3) and 1/2Nx (*n* = 4) DEK rats at 28 days after surgery, and right (R) kidneys of Sham operated (*n* = 5) and 1/2Nx (*n* = 6) DEK rats at 84 days after surgery. Relative kidney weights represent the ratios of kidney weights (g) to body weights (kg). Data are presented as mean ± SE. ** *p* < 0.01, *** *p* < 0.001 between the two groups.

**Table 2:** Food intake, water intake, and urinary volume of Sham operated and 1/2Nx DEK rats.

	28 Days		84 Days	
	Sham (*n* = 3)	1/2Nx (*n* = 5)	Sham (*n* = 3)	1/2Nx (*n* = 5)
Food intake (g/kg)	107.6 ± 13.8	46.8 ± 6.7 **	104.0 ± 14.1	62.1 ± 10.3 *
Water intake (g/kg)	371.5 ± 68.9	74.2 ± 8.4 **	397.5 ± 94.7	185.0 ± 60.1
Urinary volume (mL/kg)	281.0 ± 76.9	44.3 ± 5.3 **	362.6 ± 99.0	150.2 ± 51.3

Food intake (g), water intake (g), and urinary volume (mL) relative to body weight (kg) of Sham operated and 1/2Nx DEK rats for 24 h measured 28 and 84 days after surgery. Data are presented as mean ± SE. * *p* < 0.05, ** *p* < 0.01 between the two groups.

**Table 3:** Biochemical parameters in plasma of Sham operated and 1/2Nx DEK rats.

	28 Days		84 Days	
	Sham (*n* = 3)	1/2Nx (*n* = 5)	Sham (*n* = 3)	1/2Nx (*n* = 5)
Tcho (mg/dL)	77.7 ± 3.1	103.0 ± 4.4 **	76.7 ± 2.1	109.7 ± 5.1 **
Cre (mg/dL)	0.22 ± 0.03	0.35 ± 0.03 *	0.30 ± 0.02	0.55 ± 0.06 *
BUN (mg/dL)	16.5 ± 1.0	18.5 ± 0.5	22.3 ± 0.7	26.3 ± 2.4
Na (mEq/L)	136.7 ± 0.3	140.2 ± 0.8 *	139.0 ± 0.6	140.7 ± 0.6
K (mEq/L)	4.5 ± 0.2	4.5 ± 0.3	4.3 ± 0.1	4.9 ± 0.2
Cl (mEq/L)	95.7 ± 0.8	99.6 ± 1.2	95.0 ± 0.0	98.0 ± 1.5
Alb (g/dL)	3.6 ± 0.1	3.7 ± 0.1	2.9 ± 0.1	3.4 ± 0.1 *
TP (g/dL)	5.00 ± 0.25	5.54 ± 0.23	5.17 ± 0.09	5.38 ± 0.05 *

Plasma biochemical parameters in Sham operated and 1/2Nx DEK rats 28 and 84 days after surgery. Data are presented as mean ± SE. * *p* < 0.05, ** *p* < 0.01 between the two groups.

**Table 4:** Biochemical parameters in urine of Sham operated and 1/2Nx DEK rats.

	28 Days		84 Days	
	Sham (*n* = 3)	1/2Nx (*n* = 5)	Sham (*n* = 3)	1/2Nx (*n* = 5 or 6)
Cre (mg/kg)	49.1 ± 6.1	56.0 ± 7.2	78.1 ± 24.2	47.6 ± 1.5
UN (mg/kg)	1135.1 ± 235.9	658.1 ± 139.0	2034.6 ± 910.8	788.1 ± 123.5 *
Na (mEq/kg)	8.6 ± 2.0	4.4 ± 0.8	9.8 ± 1.3	6.0 ± 1.3
K (mEq/kg)	14.2 ± 3.2	7.6 ± 1.4	16.5 ± 2.3	10.1 ± 1.8
Cl (mEq/kg)	10.5 ± 2.2	6.9 ± 1.6	12.6 ± 1.5	6.1 ± 1.5 *
Protein (mg/kg)	132.5 ± 34.3	29.0 ± 11.3 *	146.9 ± 33.1	101.6 ± 42.0
Glu (mg/kg)	23,143.4 ± 7730.6	39.7 ± 13.1 *	31,603.7 ± 7983.1	10,918.6 ± 6777.3

Twenty-four-hour urinary excretion of Cre, UN, Na-K-Cl, protein, and Glu relative to body weight (kg) of Sham operated and 1/2Nx DEK rats 28 and 84 days after surgery. Data are presented as mean ± SE. * *p* < 0.05 between the two groups.

**Table 5:** Creatinine, sodium, and glucose clearances and fraction excretions in Sham operated and 1/2Nx DEK rats.

	28 Days		84 Days	
	Sham (*n* = 3)	1/2Nx (*n* = 5)	Sham (*n* = 3)	1/2Nx (*n* = 5 or 6)
Ccre	15.74 ± 2.69	11.60 ± 2.11	19.10 ± 7.33	6.33 ± 0.68 *
Cna (×10^2^)	4.28 ± 0.98	2.16 ± 0.39	4.88 ± 0.65	2.95 ± 0.65
Cglu	4.28 ± 1.57	0.19 ± 0.06 *	3.93 ± 0.66	1.48 ± 0.76
FEna	0.27 ± 0.02	0.19 ± 0.02 *	0.31 ± 0.07	0.49 ± 0.09
FEglu	25.57 ± 4.80	1.70 ± 0.45 *	24.91 ± 6.55	20.93 ± 9.27

Clearances and fractional excretions of Cre, Na, and Glu by Sham operated and 1/2Nx DEK rats 28 and 84 days after surgery. Data are presented as mean ± SE. * *p* < 0.05 between the two groups.

## Data Availability

Data is contained within the article.

## Supplementary Materials

The following are available online at https://www.mdpi.com/article/10.3390/biology10070633/s1, Table S1: Kidney weights before surgery (Pre) and 28 and 84 days after surgery in sham operated and 1/2Nx Wistar rats, Table S2: Food intake, water intake and urinary volume of sham operated and 1/2Nx Wistar rats., Table S3: Creatinine, sodium and glucose clearances and fraction excretions in sham operated and 1/2Nx Wistar rats., Table S4: PCNA positive cells per unit area in sham operated and 1/2Nx Wistar rats at 84 days after surgery.

## References

[1] International Diabetes Federation (2019). IDF Diabetes Atlas, 9th ed.

[2] American Diabetes Association (2010). Diagnosis and Classification of Diabetes Mellitus. Diabetes Care.

[3] Wilding J.P. (2014). The role of the kidneys in glucose homeostasis in type 2 diabetes: Clinical implications and therapeutic significance through sodium glucose co-transporter 2 inhibitors. Metabolism.

[4] Hinnen D. (2013). The Role of the Kidney in Hyperglycemia. J. Cardiovasc. Nurs..

[5] Gerich J.E. (2010). Role of the kidney in normal glucose homeostasis and in the hyperglycaemia of diabetes mellitus: Therapeutic implications. Diabet. Med..

[6] Hsia D.S., Grove O., Cefalu W.T. (2016). An update on sodium-glucose co-transporter-2 inhibitors for the treatment of diabetes mellitus. Curr. Opin. Endocrinol. Diabetes Obes..

[7] Heerspink H.J.L., Stefansson B.V., Chertow G.M., Correa-Rotter R., Greene T., Hou F.-F., Lindberg M., McMurray J., Rossing P., Toto R. (2020). Rationale and protocol of the Dapagliflozin and Prevention of Adverse outcomes in Chronic Kidney Disease (DAPA-CKD) randomized controlled trial. Nephrol. Dial. Transplant..

[8] Alsahli M., Gerich J.E. (2017). Renal glucose metabolism in normal physiological conditions and in diabetes. Diabetes Res. Clin. Pract..

[9] Rabkin R., Ryan M.P., Duckworth W.C. (1984). The renal metabolism of insulin. Diabetologia.

[10] Gerich J.E., Meyer C., Woerle H.J., Stumvoll M. (2001). Renal Gluconeogenesis: Its importance in human glucose homeostasis. Diabetes Care.

[11] Meyer C., Dostou J.M., Welle S.L., Gerich J.E. (2002). Role of human liver, kidney, and skeletal muscle in postprandial glucose homeostasis. Am. J. Physiol. Metab..

[12] Meyer C., Stumvoll M., Nadkarni V., Dostou J., Mitrakou A., Gerich J. (1998). Abnormal renal and hepatic glucose metabolism in type 2 diabetes mellitus. J. Clin. Investig..

[13] Sochor M., Kunjara S., Greenbaum A.L., McLean P. (1986). Renal hypertrophy in experimental diabetes. Effect of diabetes on the pathways of glucose metabolism: Differential response in adult and immature rats. Biochem. J..

[14] Satriano J. (2007). Kidney growth, hypertrophy and the unifying mechanism of diabetic complications. Amino Acids.

[15] Seyer-Hansen K. (1983). Renal hypertrophy in experimental diabetes mellitus. Kidney Int..

[16] Douglas-Denton R.N., McNamara B.J., Hoy W.E., Hughson M.D., Bertram J.F. (2006). Does Nephron Number Matter in the Development of Kidney Disease?. Ethn Dis..

[17] Domon A., Katayama K., Tochigi Y., Suzuki H. (2019). Characterization of Novel Nonobese Type 2 Diabetes Rat Model with Enlarged Kidneys. J. Diabetes Res..

[18] Suzuki H., Suzuki K. (1995). Pathophysiology and postnatal pathogenesis of hypoplastic kidney (hpk/hpk) in the male hypogonadic mutant rat (hgn/hgn). J. Vet. Med Sci..

[19] Suzuki H., Tokuriki T., Saito K., Hishida A., Suzuki K. (2005). Glomerular hyperfiltration and hypertrophy in the rat hypoplastic kidney as a model of oligomeganephronic disease. Nephrol. Dial. Transplant..

[20] Katsuda Y., Kemmochi Y., Maki M., Sano R., Toriniwa Y., Ishii Y., Miyajima K., Kakimoto K., Ohta T. (2014). Effects of Unilateral Nephrectomy on Renal Function in Male Spontaneously Diabetic Torii Fatty Rats: A Novel Obese Type 2 Diabetic Model. J. Diabetes Res..

[21] Yasuda H., Amakasu K., Tochigi Y., Katayama K., Suzuki H. (2016). Renal Function and Hematology in Rats with Congenital Renal Hypoplasia. Comp. Med..

[22] Domon A., Katayama K., Sato T., Tochigi Y., Tazaki H., Suzuki H. (2021). Empagliflozin ameliorates symptoms of diabetes and renal tubular dysfunction in a rat model of diabetes with enlarged kidney (DEK). PLoS ONE.

[23] Obineche E.N., Mensah-Brown E., Chandranath S.I., Ahmed I., Naseer O., Adem A. (2001). Morphological Changes in the Rat Kidney Following Long-Term Diabetes. Arch. Physiol. Biochem..

[24] Schwieger J., Fine L.G. (1990). Renal hypertrophy, growth factors, and nephropathy in diabetes mellitus. Semin. Nephrol..

[25] Christiansen J.S., Gammelgaard J., Frandsen M., Parving H.-H. (1981). Increased kidney size, glomerular filtration rate and renal plasma flow in short-term insulin-dependent diabetics. Diabetologia.

[26] Amakasu K., Suzuki K., Suzuki H. (2009). The Unilateral Urogenital Anomalies (UUA) Rat: A New Mutant Strain Associated with Unilateral Renal Agenesis, Cryptorchidism, and Malformations of Reproductive Organs Restricted to the Left Side. Comp. Med..

[27] Zimanyi M.A., Hoy W.E., Douglas-Denton R.N., Hughson M.D., Holden L.M., Bertram J.F. (2009). Nephron number and individual glomerular volumes in male Caucasian and African American subjects. Nephrol. Dial. Transplant..

[28] Duckworth W.C., Bennett R.G., Hamel F.G. (1998). Insulin Degradation: Progress and Potential. Endocr. Rev..

[29] Swe M.T., Pongchaidecha A., Chatsudthipong V., Chattipakorn N., Lungkaphin A. (2019). Molecular signaling mechanisms of renal gluconeogenesis in nondiabetic and diabetic conditions. J. Cell. Physiol..

[30] Neumiller J.J., Alicic R.Z., Tuttle K. (2017). Therapeutic Considerations for Antihyperglycemic Agents in Diabetic Kidney Disease. J. Am. Soc. Nephrol..

[31] Jørgensen M.B., Hornum M., Van Hall G., Bistrup C., Hansen J.M., Mathiesen E.R., Feldt-Rasmussen B. (2016). The impact of kidney transplantation on insulin sensitivity. Transpl. Int..

[32] Levine D.Z., Iacovitti M., Robertson S.J. (2008). Modulation of single-nephron GFR in the db/db mouse model of type 2 diabetes mellitus. II. Effects of renal mass reduction. Am. J. Physiol. Integr. Comp. Physiol..

[33] Kato Y., Ohno Y., Hayashi M., Suzawa T., Shibagaki K., Sasaki T., Saruta T. (2005). Divergent Effects of Unilateral and Subtotal Ne-phrectomy on Insulin Sensitivity in Rats. Ren Fail..

[34] Sugaya K., Ogawa Y., Hatano T., Koyama Y., Miyazato T., Naito A., Yonou H., Kagawa H. (2000). Compensatory renal hypertrophy and changes of renal function following nephrectomy. Hinyokika kiyo. Acta Urol. Jpn..

[35] Dicker S.E., Shirley D.G. (1971). Mechanism of compensatory renal hypertrophy. J. Physiol..

[36] Thabet M.A.E.H., Salcedo J.R., Chan J.C.M. (1993). Hyperlipidemia in childhood nephrotic syndrome. Pediatr. Nephrol..

[37] Uil M., Scantlebery A., Butter L.M., Larsen P.W.B., De Boer O.J., Leemans J.C., Florquin S., Roelofs J.J.T.H. (2018). Combining streptozotocin and unilateral nephrectomy is an effective method for inducing experimental diabetic nephropathy in the ‘resistant’ C57Bl/6J mouse strain. Sci. Rep..

[38] Nakajima T., Miyaji T., Kato A., Ikegaya N., Yamamoto T., Hishida A. (1996). Uninephrectomy reduces apoptotic cell death and enhances renal tubular cell regeneration in ischemic ARF in rats. Am. J. Physiol. Physiol..

[39] Nakazawa T., Kasahara K., Ikezaki S., Yamaguchi Y., Edamoto H., Nishimura N., Yahata M., Tamura K., Kamata E., Ema M. (2009). Renal Tubular Cyst Formation in Newborn Rats Treated with p-Cumylphenol. J. Toxicol. Pathol..

