# Supplementary material for: Characterization of Enlarged Kidneys and Their Potential for Inducing Diabetes in DEK Rats
Source: Biology (Basel). 2021 Jul 8;10(7):633. doi: 10.3390/biology10070633 (PMC8301011; doi:10.3390/biology10070633)
Supplement: Supplementary file 1 [file biology-10-00633-s001.zip › biology-1268554-supplementary.pdf]

biology MDPI

Supporting Information

Male Wistar rats at 16 weeks of age were subjected to uninephrectomy or sham operation. Rats were sacrificed after measurement of metabolic parameters at twenty-eight or eighty-four days after surgery. All procedures are the same as described in the main text.

Table S1. Kidney weights before surgery (Pre) and 28 and 84 days after surgery in sham operated and 1/2Nx Wistar rats.

	Kidney weight (g)			Relative kidney weight (g/kg)		
	Pre/L side	28 days/R side	84 days/R side	Pre/L side	28 days/R side	84 days/R side
Sham	-	1.6±0.0	1.8±0.2	-	2.9±0.0	2.7±0.2
1/2Nx	1.9±0.18	1.9±0.1	2.3±0.1	4.3±0.4	4.1±0.0***	3.7±0.2

The average kidney weights of left (L) kidneys removed from 1/2Nx Wistar rats (Pre, n = 10), right (R) kidneys of sham operated (n = 3) and 1/2Nx (n = 3) Wistar rats at 28 days after surgery, and right (R) kidneys of Sham operated (n = 3) and 1/2Nx (n = 7) Wistar rats at 84 days after surgery. Relative kidney weights represent the ratios of kidney weights (g) to body weights (kg). Data are presented as mean ± SE. *** $P< 0.001$ between the two groups.

Table S2. Food intake, water intake and urinary volume of sham operated and 1/2Nx Wistar rats.

	28 days		84 days	
	Sham (n=4)	1/2Nx (n=3)	Sham (n=3)	1/2Nx (n=6)
Food intake (g/kg)	58.6±2.2	69.6±7.8	38.8±9.6	48.1±1.6
Water intake (g/kg)	70.9±5.6	85.2±6.7	49.1±12.4	52.4±4.9
Urinary volume (ml/kg)	28.7±4.9	31.1±7.7	19.4±2.2	18.9±2.7

Food intake (g), water intake (g) and urinary volume (ml) relative to body weight (kg) of sham operated and 1/2Nx Wistar rats for 24 hours measured 28 and 84 days after surgery. Data are presented as mean ± SE.

Table S3. Creatinine, sodium and glucose clearances and fraction excretions in sham operated and 1/2Nx Wistar rats.

	28 days		84 days	
	Sham (n=4)	1/2Nx (n=3)	Sham (n=3)	1/2Nx (n= 6)
Ccre	9.09±1.34	7.38±1.14	6.39±1.03	5.10±0.53
Cna (×10²)	2.76±0.43	2.82±0.52	1.13±0.22	1.75±0.13*
Cglu	0.31e-2±0.06e-2	0.19e-2±0.04e-2	0.10e-2±0.01e-2	0.18e-2±0.03e-2
FEna	0.30±0.01	0.39±0.04	0.18±0.01	0.36±0.03**
FEglu	0.03±0.04e-1	0.03±0.04e-1	0.02±0.04e-1	0.04±0.05e-1

Clearances and fractional excretions of Cre, Na, and Glu by sham operated and 1/2Nx Wistar rats 28 and 84 days after surgery. Data are presented as mean ± SE. * $P< 0.05$, ** $P< 0.01$ between the two groups.

Table S4. PCNA positive cells per unit area in sham operated and 1/2Nx Wistar rats at 84 days after surgery.

	Sham (n=3)	1/2Nx (n=3)
Outer cortex	1.70±0.16	5.43±0.31***
Juxtamedullary cortex	2.17±0.14	9.10±.47***

Number of PCNA positive cells in renal tubules in unit area. Data are presented as mean ± SE. *** $P< 0.001$ between the two groups.
